# Tauvid™: The First FDA-Approved PET Tracer for Imaging Tau Pathology in Alzheimer’s Disease

**DOI:** 10.3390/ph14020110

**Published:** 2021-01-30

**Authors:** Caitlin V. M. L. Jie, Valerie Treyer, Roger Schibli, Linjing Mu

**Affiliations:** 1 Center for Radiopharmaceutical Sciences, Department of Chemistry and Applied Biosciences, ETH Zürich, 8093 Zurich, Switzerland; caitlin.jie@pharma.ethz.ch (C.V.M.L.J.); roger.schibli@psi.ch (R.S.); 2 Department of Nuclear Medicine, University Hospital Zurich, 8091 Zurich, Switzerland; valerie.treyer@usz.ch

**Keywords:** tauvid™, [^18^F]flortaucipir, alzheimer’s disease, tau neurofibrillary tangles (NFTs), PET

## Abstract

Tauvid has been approved by the U.S. Food and Drug Administration (FDA) in 2020 for positron emission tomography (PET) imaging of adult patients with cognitive impairments undergoing evaluation for Alzheimer’s disease (AD) based on tau pathology. Abnormal aggregation of tau proteins is one of the main pathologies present in AD and is receiving increasing attention as a diagnostic and therapeutic target. In this review, we summarised the production and quality control of Tauvid, its clinical application, pharmacology and pharmacokinetics, as well as its limitation due to off-target binding. Moreover, a brief overview on the second-generation of Tau PET tracers is provided. The approval of Tauvid marks a step forward in the field of AD research and opens up opportunities for second-generation tau tracers to advance tau PET imaging in the clinic.

## 1. Introduction

On the 28 May 2020, the U.S. Food and Drug Administration (FDA) approved Tauvid—a radioactive tracer—for positron emission tomography (PET) imaging of tau pathology in Alzheimer’s disease (AD) [1]. AD is a neurodegenerative disorder and the leading cause of dementia. According to the Alzheimer’s Disease International, it is estimated that over 50 million people worldwide have dementia, which is set to increase to over 150 million by 2050 [2]. Akin to many other neurodegenerative diseases, AD pathology is closely related to the accumulation of one or more folded or misfolded proteins. Two of the main hallmarks of AD are amyloid-β (Aβ) and tau, which cause the spread of extracellular Aβ plaques and intracellular tau neurofibrillary tangles (NFTs), respectively. Due to their evident appearance in AD patients, these have been the main focus in AD research as drug targets, as well as diagnostic targets [3].

PET is a noninvasive imaging technique that enables identification and monitoring of cellular and molecular changes within the body [4]. It uses trace amounts of radioactive substance, the so-called radiotracer, to image functions without disturbing the biological process in question. The classic Lipinski’s “rule-of-5” [5] for central nervous system (CNS) drugs is also valid for the majority of successful PET tracers for brain imaging [6]. A CNS PET tracer should provide sufficient permeability of the blood–brain barrier, favourable blood–brain distribution, appropriate kinetics in brain, and absence of brain-penetrant radiometabolites. To achieve a high specific PET signal in vivo, the radioligand is expected to possess high binding affinity towards the target and display low nonspecific binding and high target selectivity [7].

Aβ tracers have been thoroughly investigated and the related research has grown exponentially in recent years. Between 2012 and 2014, [^18^F]florbetapir (Amyvid™), [^18^F]flutemetamol (Vizamyl™), and [^18^F]florbetaben (Neuroceq™) were consecutively approved by the FDA and the European Medicines Agency for imaging Aβ plaques in AD patients [8]. These PET tracers have greatly impacted the diagnosis of AD patients in the clinic and can assist in evaluating patients with cognitive impairment and dementia. Whilst most AD patients are positive for Aβ as indicated by Aβ PET tracers, there is still an unmet clinical need for a reliable, sensitive, and noninvasive tool to monitor disease progression as Aβ plaque deposition and cognitive impairment are poorly correlated in AD [9]. To further improve diagnostics and monitoring of disease progression, research has shifted increasingly towards the other main pathology: tau tangles.

**Figure 1:**
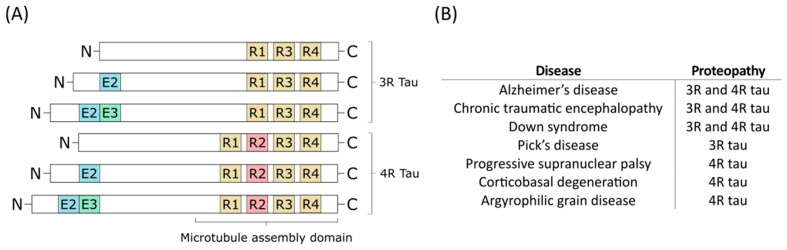
(**A**) The six different tau isoforms of an adult human. Exon 2 (E2), exon 3 (E3), and exon 10 (R2) are indicated in blue, green, and red, respectively. Alternative splicing creates the different isoforms which can be divided into 3-repeat (3R) tau and 4-repeat (4R) tau. (**B**) Different diseases can involve only 3R tau or 4R tau, or both of them as the main proteopathy [7].

Tau is a neuronal microtubule-associated protein that promotes microtubule self-assembly by tubulin and modulates the stability of axonal microtubules. The brain of an adult human contains six main isoforms of tau, where they are generated by alternative splicing of exons 2, 3, and 10. These isoforms are further categorised by whether they have a three or four carboxy-terminal repeat domains—which are referred to as 3R or 4R tau isoforms, respectively (Figure 1) [10].

**Figure 2:**
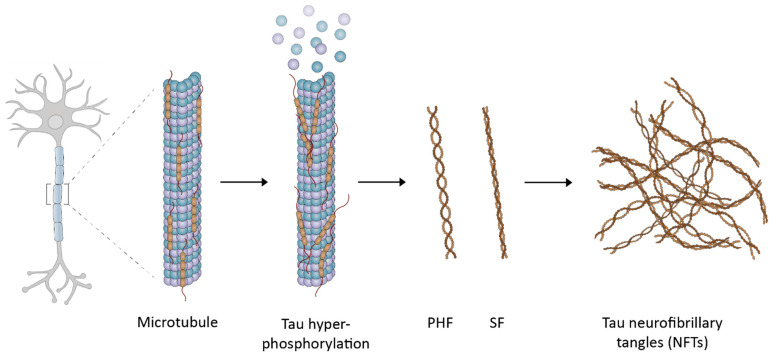
The development of tau neurofibrillary tangles (NFTs): tau proteins self-aggregate to form loosely intertwined paired helical filaments (PHFs) and the tightly wrapped straight filaments (SFs), which then leads to the formation of NFTs.

Conformational changes in tau proteins but also hyperphosphorylation contributes to the formation of tau aggregates that eventually form NFTs (Figure 2) [11]. Typically, tau pathology first appears in the entorhinal cortex before it spreads further into the hippocampus, parahippocampal gyrus, temporal lobes, and the rest of the neocortex [12]. The distribution of NFTs can be classified into four different stages: (1) B0, no NFTs; (2) B1 (Braak stages I/II), predominantly in the entorhinal cortex; (3) B2 (Braak stages III/IV), abundant in hippocampus, amygdala, and some in the association cortex; (4) B3 (Braak stages V/VI), widely distributed throughout the neocortex [13]. Tau pathology has been reported to be closely associated with cognitive decline—particularly in the cortical regions—which makes it an attractive diagnostic target to monitor cognitive decline [14,15,16,17].

Compared to Aβ imaging, the regional distribution of tau deposits in the brain is expected to have better correlation with disease progression. For example, NFTs but not amyloid deposits have been shown to be related to neuronal loss in AD [14]. Brier et al. also suggest that tau pathology to be more closely linked to cognitive dysfunction than Aβ imaging by showing a close relationship between tau PET imaging and AD disease stage [16]. Due to its close relationship with disease progression, tau has recently been considered as a potential therapeutic target to reduce or delay AD progression. Tau-kinase inhibitors, acetylation inhibitors, microtubule stabilisers, aggregation inhibitors, and monoclonal anti-tau antibodies are being tested for inhibiting or decelerating different aspects of tau pathology [18]. Among them, several tau-targeted immunotherapies have shown promise in preclinical studies and reached clinical trials [11]. Therefore, tau imaging could be a powerful tool to classify patients in clinical trials as well as to aid in treating patients at an appropriate time to halt further disease progression.

**Figure 3:**
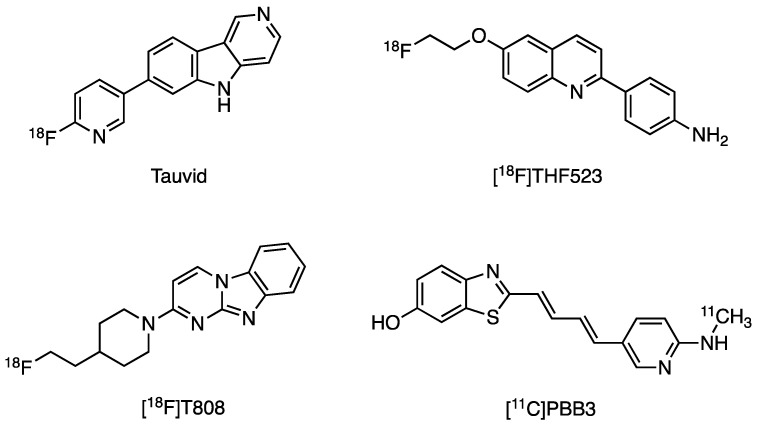
First-generation tau positron emission tomography (PET) tracers, including Tauvid.

Apart from Tauvid, there are several other first-generation tau tracers (Figure 3) that were developed but encountered some major limitations. The quinolone-based tracers such as [^18^F]THK523 showed high nonspecific binding in white matter [19]. [^11^C]PBB3, on the other hand, is light-sensitive, and its radioactive metabolite could enter the brain which complicates quantification of tau pathology [20]. Although [^18^F]T808 showed favourable kinetic properties, the high bone uptake revealed the problem of defluorination [21]. Thus, compared to the other first-generation tau tracers, Tauvid has shown some strength by overcoming these reported problems and therefore received approval from the FDA for clinical usage. However, Tauvid PET imaging is still facing several challenges in terms of off-target binding and early detection of AD which limit its application in clinical use (vide infra) [22]. The current ongoing clinical trials with the second generation of Tau PET tracers have great potential to provide high specific signals and therefore will facilitate diagnosis of tau-related neurodegenerative diseases (reported in Section 4). 

In addition to being a PET imaging biomarker, tau has also been investigated as a fluid biomarker, which may enable screening of patients before undergoing PET imaging [23]. Cerebral spinal fluid (CSF) phosphorylated tau (p-tau) levels do increase at an early disease stage but appear to reach a plateau or even decrease at later stages [24,25], which limits following disease progression using CSF p-tau. Although still in its infancy, plasma p-tau appears to be more promising and may allow early AD patient selection [26,27]. One drawback of fluid biomarkers is the inability to follow brain-specific changes and the spatial distribution of the pathology, which make disease staging more challenging compared to brain imaging, thus suggesting that noninvasive tau PET imaging is still an irreplaceable tool for estimating density and distribution of tau pathology in AD.

## 2. Chemical Overview

### 2.1. Names and Structure

Tauvid is an ^18^F-labelled benzimidazole pyridine derivative which was discovered by Siemens Molecular Imaging Biomarker Research (recently acquired by Avid/Lilly). It was selected and developed by screening a various chemical class of compounds using isolated PHF-tau from postmortem AD brain tissues and intact human brain tissue sections [28]. Tauvid is also known as [^18^F]Flortaucipir, [^18^F]AV-1451, and [^18^F]T807. Its IUPAC name is 7-(6-[^18^F]fluoropyridine-3-yl)-5H-pyrido [4,3-b]indole. The molecular weight is 262.27, and the structural formula is illustrated in Figure 3.

### 2.2. Fluorine-18

Fluorine-18 has a half-life of 109.8 min with a high positron decay ratio (97%) and a low positron energy (maximum 0.635 MeV). Fluorine-18 is favourable for PET due to its positron energy that results in a short diffusion range of <2.4 mm in water [29]. The photons used for diagnostic imaging are the coincident pair of 511 keV gamma photons that resulted from the interaction of the emitted positron with an electron.

No-carrier-added ^18^F-fluoride is produced in a cyclotron by proton irradiation of oxygen-18 enriched water target (^18^O(p,n)^18^F nuclear reaction). The ^18^F-flouride is obtained as an aqueous solution and, due to its high charge density, is strongly hydrated and inactivated for nucleophilic reactions. Therefore, the produced ^18^F-fluoride is routinely trapped on solid-phase extraction cartridge, followed by elution with a solution of Kryptofix 222 (K_222_)/K_2_CO_3_ and successive azeotropic drying with acetonitrile. The addition of phase transfer catalysts such as Kryptofix 222 can enhance the solubility and nucleophilicity of fluoride ions in organic solvents. The dried ^18^F-fluoride displays increased nucleophilicity and can be used in various aliphatic and aromatic nucleophilic substitution reactions. 

### 2.3. Manufacturing and Quality Criteria.

**Scheme 1:**
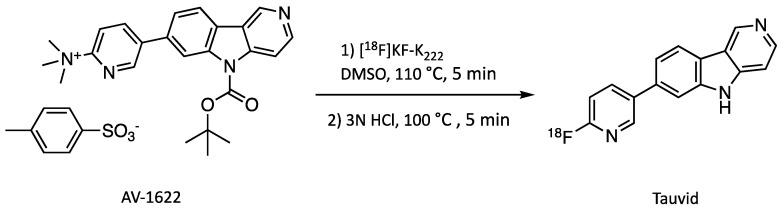
Radiosynthesis of Tauvid.

Originally, the radiosynthesis of Tauvid was performed using the precursor 7-(6-nitropyridin-3-yl)-5H-pyrido[4,3-b]indole, it has similar lipophilicity with the fluorinated compound Tauvid and complicates the purification process. After nucleophilic ^18^F-substitution with the nitro group on the pyridine ring, a second step is carried out using iron powder/formic acid to reduce the nitro group on the remaining precursor to the respective 2-amino-pyridine derivative for facilitating the separation by HPLC. This step requires a separate vial offline from the automated synthesis unit which is not readily adaptable to commercial radiosynthesis platforms and poses a limitation for advancing large-scale multicentre trials and widespread use. [28]. An improved precursor AV-1622, an *N*-Boc-protected trimethylammonium precursor, was used later (Scheme 1) to facilitate the radiosynthesis of Tauvid by improving the solubility, reactivity, separability, and yield [30]. 

Tauvid should be produced with an automated synthesis module in a laboratory according to Good Manufacturing Practice (GMP). The radiolabelling was accomplished by reacting the precursor compound with no-carrier-added [^18^F]fluoride. After deprotection and neutralisation, the crude product was purified by semipreparative HPLC and formulated by a C-18 light cartridge. The sterile filtration and filling were accomplished under aseptic conditions. The excipients of injected Tauvid include less than 10% ethanol in 0.9% sodium chloride injection (USP) and sodium ascorbate to suppress radiolysis.

Several quality control procedures should be conducted before delivery to the clinics [31]. The product is visually inspected and should be clear and colourless. The radiochemical identity and radiochemical and chemical purity were evaluated by HPLC equipped with a radioactivity detector. The molar activity was determined by using a standard mass calibration curve, where a molar activity ≥300 mCi/μmol is acceptable. The amount of residue solvents was determined by gas chromatography, where the acceptable amount of acetonitrile was ≤400 ppm, methanol ≤3000 ppm, and ethanol ≤10% of total volume. The pH of the product should be between a pH of 4.5 and 8.5. By thin layer chromatography, the residual Kryptofix 222 was assessed, where an acceptable level was <50 µg/mL. The radionuclide identity was confirmed by calculating the half-life, where the acceptable specification was a half-life between 105 and 115 min. Furthermore, a sterile filter integrity test, endotoxin testing, and sterility testing were performed, where endotoxin levels ≤11 endotoxin units per millilitre was acceptable and where there was no growth observed during a 14-day incubation period in the sterility testing. The limits for residue solvents, amount of Kryptofix 222, and endotoxin levels are comparable to the FDA-approved ^18^F-labelled Aβ PET tracers. Expiration time of Tauvid is based on molar activity or strength with a maximum expiry of 10 h after end of synthesis. Each package of Tauvid injection included a sterile apyrogenic syringe or sterile apyrogenic sealed glass vial containing Tauvid injection, a surrounding protective lead shield canister, and an outside delivery case.

## 3. Medicinal and Pharmaceutical Overview

### 3.1. Clinical Indication

The clinical indication of Tauvid is for estimating the density and distribution of NFTs in the brain of adult patients with cognitive impairments who are being evaluated for AD by PET [32].

Tauvid is explicitly not indicated in the labelling for the evaluation of patients for chronic traumatic encephalopathy (CTE). The differences in tau conformation and distribution may limit the binding of Tauvid and is therefore not indicated for CTE at the moment [32]—some further studies are discussed later.

### 3.2. Application

There are no known contraindications of the usage of Tauvid. Pregnant or lactating females have been excluded from all Tauvid studies due to the risk of radiation. The recommended amount of radioactivity to administer Tauvid for PET imaging is 370 MBq (10 mCi) as an intravenous bolus, followed by a normal saline flush prior to imaging. Lower doses (around 200 MBq) may be administered depending on the objectives for the PET scan and sensitivity of the PET scanner. The maximum mass dose of nonradioactive Tauvid should not exceed 20 µg. Image acquisition should start approximately 80 min after Tauvid administration to obtain a 20-minute PET image of the patient.

Tauvid might have a potential cardiotoxicity as it had an IC_50_ value of 0.610 μM in the in vitro hERG assay. Nevertheless, the safety margin is at least 42-fold when given a 20 µg dose [33], and therefore no cardiotoxicity is expected. Moreover, in vivo cardiovascular evaluation in dogs showed no evidence of QT prolongation. QT prolongation is a measure of delayed ventricular repolarisation, which means the heart muscle takes longer than normal to recharge between beats. Nonetheless, clinical trials exclude subjects with a history of risk factors for *torsades de pointes* and subjects taking drugs known to prolong the QT interval until more human cardiovascular safety data are available [33].

### 3.3. Pharmacology, Pharmacokinetics, and Pharmacodynamics

Tauvid is reported to have high affinity to immunopurified PHF-tau from postmortem human AD brain tissue with a K_D_ value of 0.68 nM by homologous competition [33], and a K_D_ value 0.57 nM with a B_max_ of 309 pmol/mg protein as determined by a saturation binding experiment [32,33]. Using postmortem sections of the frontal lobe region of AD patients, a K_D_ value of 15 nM was determined in an in vitro autoradiography-based saturation binding study [34]. Tauvid was reported to specifically bind to native tau aggregates in human brain sections, whereas in vitro autoradiography and immunostaining studies showed that binding correlates with tau but not with Aβ. Moreover, no K_D_ could be determined for Aβ, supporting the selectivity of Tauvid for PHF-tau over Aβ [28]. Tauvid was assessed against a panel of CNS receptors, ion channels, transporters, enzymes, and human tissues by competitive binding and functional assays. For most of these targets, <50% inhibition of specific binding was achieved at concentration of 10 µM Tauvid, except for norepinephrine transporter, monoamine transporter (VMAT2), polyamine site on the glutamate receptor, µ-opiate receptor, and acetylcholinesterase. IC_50_ values were determined for the norepinephrine transporter, VMAT2, and polyamine site of the glutamate receptor: 2.2, 0.4, and 2.7 μM, respectively [28]. Despite that some inhibition is observed, these are far above the concentrations used for PET imaging.

Tauvid has a measured log*P* value of 1.67 and was found to efficiently cross the blood–brain barrier with a rapid brain penetration and fast washout [28]. After intravenous injection of Tauvid, it is distributed throughout the body with <10% of injected radioactivity present in the blood 5 min after administration, demonstrating a rapid blood clearance [32]. The clearance of Tauvid primarily occurs by hepatobiliary and renal excretion. Tauvid is metabolised by CYP1A2, CYP2C8, and CYP3A4 [33] and polar metabolites are observed by radio-HPLC [35]. The radiation dosimetry of Tauvid have been evaluated from nine subjects from a clinical trial study and the effective radiation dose is approximated to be 8.7 mSv after administration of 370 MBq of Tauvid to an adult (70 kg) [32], which is comparable to the FDA-approved AD PET tracers such as Amyvid and Vizamyl (Table 1). The highest dose was observed in the upper large and small intestinal walls, followed by the liver and small intestine. Similar results have been found by Choi et al. [36], where the organ with the highest radiation absorbed dose was the liver.

### 3.4. Clinical Evaluation

The approval of Tauvid by the FDA was based on the results of two clinical trials. The first was an open label, multicentre study (Avid Radiopharmaceuticals, Philadelphia, PA, USA; NCT02516046) where the brain distribution and retention of Tauvid were evaluated compared to florbetapir PET amyloid status, type of diagnosis, age, and cognitive function. The PET imaging results were in line with the hypothesis that Aβ and tau pathology may start independently. However, the spreading of tau is associated or dependent on amyloid accumulation [39]. Furthermore, they corroborate the potential association between cortical tau and cognitive impairment and neuronal dysfunction. In a subsequent clinical study (Avid Radiopharmaceuticals, NCT03901092), the accuracy and reliability of Tauvid was assessed for PET scan interpretation. The PET scans of previously acquired studies (Avid Radiopharmaceuticals, NCT02516046 and NCT02016560) were used to evaluate the interpretation of the scans by independent, blinded readers [40]. The PET results from the completed clinical studies indicated little focal cortical retention of Tauvid in either young or older cognitively normal volunteers. Although, older cognitively normal volunteers did show frequent retention in the mesial temporal lobes and some also in the brainstem or striatum. In subjects with mild cognitive impairment and AD, retention seems to spread from the mesial temporal lobes to isocortical areas, which appears to go in parallel with the Braak staging [13]. Altogether, a total of 59 subjects were injected with Tauvid in the completed clinical trials prior to FDA approval, where all reported adverse effects were mild (such as diarrhoea and headache) and all subjects have recovered. The reported musculoskeletal pain and hypertension were considered related to Tauvid administration (e.g., site injection pain), the PET imaging procedure (e.g., requirement of lying still in the PET scanner), and the time of blood pressure measurements. There were no other reports of consistent or clinically relevant changes in vital signs, laboratory values, or electrocardiography results.

In a small clinical study of 32 symptomatic AD patients, Tauvid PET imaging could predict the rate of brain atrophy as determined by structural MRI, whilst Aβ PET imaging with [^11^C]PIB was not able to estimate this [41]. Furthermore, since tau pathology is also closely related to cognitive impairment, tau PET might be a valuable tool for following tau pathology in Aβ- and tau-targeted therapies. Aβ PET imaging does not have this application due to its poor correlation with cognitive decline. Therefore, Tauvid is now also used as a supportive tool in clinical trials to monitor tau pathology during treatment with monoclonal antibody therapy targeting Aβ, such as Donanemab (Eli Lilly and Company, Indianapolis, IN, USA; NCT04437511), Solanezumab and Gantenerumab (Washington University School of Medicine, St. Louis, MO, USA; NCT01760005). This demonstrates the versatility of Tauvid as it can serve as a diagnostic tool for AD but can also support interventional clinical trials by monitoring the tau load and give insight into the disease progression of the patients also beyond AD.

## 4. Perspective

To date, Tauvid has been evaluated in patients with generally severe stages of dementia and its performance may be less accurate when looking at patients in the earlier stages of cognitive decline. A fraction of patients with clinically meaningful tau pathologies might go unnoticed as distinguishing stage B2 specifically has shown to be challenging [42]. The off-target binding of Tauvid to the choroid plexus, meninges, iron-associated regions, and neuromelanin- and melanin-containing cells likely contributes to difficult identification of these specific stages of tau pathology [43,44]. However, in the case of the choroid plexus, there is still some controversy on whether the observed binding is related to tauopathy. One study showed tangle-like structures in the epithelial cells of the choroid plexus that can be labelled with a fluorescent Tauvid derivative and a Congo red derivative, and are immunoreactive to tau-specific antibodies, implying it might be on-target binding of Tauvid [45]. On the other hand, it has been reported that it is off-target binding to leptomeningal melanocytes [46], or to choroid plexus calcifications [44]. Therefore, further target evaluation in the choroid plexus is warranted as it may be an important region to consider in tau imaging due to its proximity to the hippocampus. In an in vitro assay, Tauvid has been reported to bind to monoamine oxidases (MAO): MAO-A (K_D_ = 1.6 nM) and MAO-B (K_D_ = 21 nM) [47]. However, binding to MAO-B has been demonstrated to be not significant in vivo, making MAO-B not a likely off-target binding site in the basal ganglia [48,49].

There are some limitations in using Tauvid PET imaging in certain cases such as supranucleur palsy (PSP), patients with CTE, patients with Down syndrome, and dementia with Lewy bodies [22,50,51]. In PSP there is a substantial overlap of signals in multiple brain regions when comparing PSP patients with AD patients and healthy subjects [47]. Nonetheless, Tauvid may be still useful when comparing PSP patients with PD patients as one clinical trial reported elevated uptake of Tauvid compared to controls and PD patients [52]. For CTE, there is a modest correlation between Tauvid PET results and postmortem pathology [53]. Various studies have shown some potential of Tauvid for CTE but it still has its limitations [53,54,55,56]. Further studies with second-generation tracers might elucidate the use of tau PET imaging in CTE and other tau-related neurodegenerative diseases.

**Figure 4:**
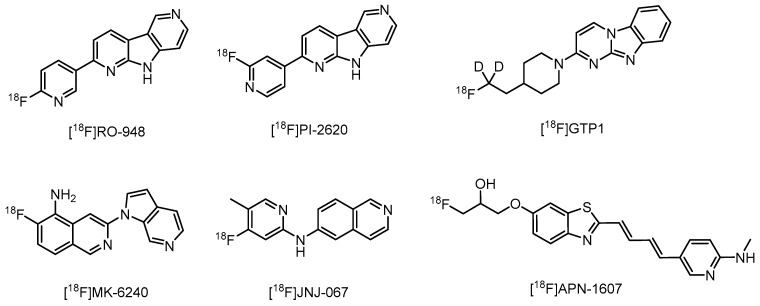
Structures of second-generation Tau PET tracers currently in clinical evaluation.

Over the last few years, significant efforts have led to the development of the second-generation tau ligands in order to overcome the challenges that first-generation tau ligands encountered (Figure 4). A number of second-generation ligands have been taken on by pharma companies and are currently under clinical evaluation, such as [^18^F]PI-2620 (AC Immune, Lausanne, Switzerland; NCT03510572), [^18^F]MK-6240 (Merck, Darmstadt, Germany; NCT03071224), [^18^F]RO-948 (Roche, Basel, Switzerland; NCT03939780), [^18^F]JNJ-067 (Janssen, Beerse, Belgium; NCT03926702), [^18^F]APN-1607 (Aprinoia Therapeutics, Taipei city, Taiwan; NCT04141150), and [^18^F]GTP1 (Genentech, South San Francisco, CA, USA; NCT04394845). Both [^18^F]RO-948 and [^18^F]PI-2620 are derivatives of Tauvid and have already shown to be safe to use and are able to distinguish AD subjects from healthy controls [57,58]. [^18^F]RO-948 has also been reported to have less off-target binding as compared to Tauvid in the known off-target regions, such as the choroid plexus and subcortical grey matter structures [59]. [^18^F]PI-2620 binds both 3R and 4R tau isoforms and has significantly reduced MAO-A binding compared to Tauvid. It was found that instead of a pyrido[4,3-b]indole scaffold, a pyrrolo[2,3-b:4,5-c’]dipyridine scaffold significantly reduces the affinity to MAO-A but retains the high affinity to tau [60]. Another second-generation tau tracer in clinical trials is [^18^F]MK-6240 (also known as [^18^F]MNI-946) (Columbia University, New York, NY, USA; NCT03373604). The preclinical studies and phase 1 clinical trial with [^18^F]MK-6240 (Merck, Darmstadt, Germany; NCT02562989) have demonstrated that there was no off-target binding in regions such as the choroid plexus that were evident with Tauvid [61,62,63]. Further clinical investigation awaits the second-generation tau tracers to evaluate their potential for imaging in Alzheimer’s disease and other tau-related diseases.

## 5. Conclusions

Tauvid is the first approved PET tracer for imaging tau pathology in AD by the FDA. It is indicated as useful for assessing the density and distribution of NFTs in the brain of adult patients with cognitive impairments who are being evaluated for AD. Tauvid has high selectivity towards tau over Aβ and is safe to be used in the clinical trials. Tauvid PET imaging can distinguish late-stage AD from cognitively normal subjects but has a limitation in evaluating earlier stages of AD. Reported off-target binding in brain regions can interfere with quantification of Tauvid uptake in the hippocampus and neighbouring mesial temporal lobe structures. The second-generation tau PET tracers are more specific with negligible off-target binding, and therefore will be more reliable to detect tau pathology. It can be expected that tau PET imaging together with Aβ PET imaging will allow a more accurate evaluation of AD patients by visualisation of the deposition of both Aβ and tau over time. Such information offers a unique opportunity to advance our knowledge on AD and can assist clinicians in deciding on a more effective treatment plan.

## Figures and Tables

**Table 1:** Radiation dose estimates of Tauvid, Amyvid, and Vizamyl. *Assumed dose weighting factor of 1 for conversion of absorbed dose to dose equivalent for ^18^F. To obtain radiation absorbed dose in rad/mCi, multiply the dose by 0.0037 = 0.0518 rad/mCi. Adapted from the labels of Tauvid [32], Amyvid [37], and Vizamyl [38].

Organ	Estimated Radiation Dose of Tauvid (µGy/MBq)	Estimated Radiation Dose of Amyvid (µGy/MBq)	Estimated Radiation Dose of Vizamyl (µGy/MBq)
Adrenal glands	14	14	13
Brain	8	10	11
Breasts	7	6	5
Gallbladder wall	38	143	287
Lower large intestine wall	25	28	42
Small intestine wall	85	66	102
Stomach wall	13	12	12
Upper large intestine wall	96	74	117
Heart wall	30	13	14
Kidneys	40	14	31
Liver	57	64	57
Lungs	34	9	16
Muscle	9	9	9
Ovaries	21	18	25
Pancreas	14	14	15
Red bone marrow	10	14	13
Osteogenic cells	12	28	11
Skin	6	6	5
Spleen	10	9	15
Testes	7	7	8
Thymus gland	9	7	6
Thyroid	7	7	6
Urinary bladder wall	38	27	145
Uterus	18	16	25
Total body	12	12	12
Effective dose (µSv/MBq)*	24	19	32
